# The Hypothetical Inclusion Membrane Protein CPSIT_0846 Regulates Mitochondrial-Mediated Host Cell Apoptosis *via* the ERK/JNK Signaling Pathway

**DOI:** 10.3389/fcimb.2021.607422

**Published:** 2021-02-26

**Authors:** Ting Tang, Haiying Wu, Xi Chen, Li Chen, Luyao Liu, Zhongyu Li, Qinqin Bai, Yuyu Chen, Lili Chen

**Affiliations:** ^1^ Department of Public Health Laboratory Sciences, College of Public Health, University of South China, Hengyang, China; ^2^ Key Laboratory of Hengyang for Health Hazard Factors Inspection and Quarantine, College of Public Health, University of South China, Hengyang, China; ^3^ Department of Infection Control, The First People’s Hospital of Yunnan Province, The Affiliated Hospital of Kunming University of Science and Technology, Kunming, China; ^4^ Department of Laboratory Medicine, The Second Affiliated Hospital of University of South China, Hengyang, China; ^5^ Institute of Pathogenic Biology, Hengyang Medical College, University of South China, Hengyang, China; ^6^ Hunan Cancer Hospital and The Affiliated Cancer Hospital of Xiangya School of Medicine, Central South University, Changsha, China

**Keywords:** *Chlamydia psittaci*, CPSIT_0846, apoptosis, mitochondria, ERK/JNK signaling pathway

## Abstract

*Chlamydia psittaci* is an important zoonotic factor associated with human and animal atypical pneumonia. Resisting host cell apoptosis is central to sustaining *Chlamydia* infection *in vivo*. *Chlamydia* can secrete inclusion membrane proteins (Incs) that play important roles in their development cycle and pathogenesis. CPSIT_0846 is an Inc protein in *C. psittaci* identified by our team in previous work. In the current study, we investigated the regulatory role of CPSIT_0846 in HeLa cell apoptosis, and explored potential mechanisms. The results showed that HeLa cells treated with CPSIT_0846 contained fewer apoptotic bodies and exhibited a lower apoptotic rate than untreated cells either with Hoechst 33258 fluorescence staining or flow cytometry with or without induction by staurosporine (STS). CPSIT_0846 could increase the phosphorylation of the extracellular signal-regulated kinases 1/2 (ERK1/2) or stress-activated protein kinases/c-Jun amino-terminal kinases (SAPK/JNK) signaling pathways, and the Bcl-2 associated X protein (Bax)/B cell lymphoma 2 (Bcl-2) ratio, levels of cleaved caspase-3/9 and cleaved Poly-ADP-ribose polymerase (PARP) were significantly up-regulated following inhibition of ERK1/2 or SAPK/JNK pathways with U0126 or SP600125. After carbonyl cyanide 3-chlorophenylhydrazone (CCCP) treatment, the mitochondrial membrane potential (MMP) of cells was significantly decreased in control group, but stable in the CPSIT_0846 treated one, and less cytochrome c (Cyt.c) was released into the cytoplasm. Inhibition of the ERK1/2 or SAPK/JNK pathway significantly decreased the JC-1 red-green fluorescence signal, and promoted Cyt.c discharge into the cytoplasm in HeLa cells treated with CPSIT_0846. In conclusion, CPSIT_0846 can regulate mitochondrial pathway-mediated apoptosis in HeLa cells by activating the ERK/JNK signaling pathway.

## Introduction

*Chlamydia psittaci* is an unique intracellular gram-negative pathogen that causes a variety of acute and chronic diseases in humans, mammals and birds, resulting in severe inflammatory damage in numerous organs and ultimately in pneumonia, conjunctivitis, and other conditions (Lin et al., 2019; Wang et al., 2019; Brokatzky et al., 2020; Gough et al., 2020). Humans can be infected by breathing in *C. psittaci* in aerosol form, or *via* contact with infected animalsand their excrement (Liu et al., 2019). *C. psittaci* usually causes flu-like symptoms in humans that may progress to sepsis, atypical pneumonia and other systemic illnesses (Collina et al., 2012; Vandendriessche et al., 2019). *C. psittaci* can also cause adnexal marginal zone lymphoma (Behdad et al., 2019). Studying the pathogenic mechanism of *C. psittaci* is very important for controlling associated diseases and promoting the economic development of the livestock industry.

Chlamydial inclusion membrane proteins (Incs) are proteins which can rearrange the inclusion membrane (IM) and thereby affect chlamydial pathogenesis (Shaw et al., 2018; Dickinson et al., 2019). The first reported Inc protein is IncA, secreted by *C. psittaci*. Then its homologues were found to exist in other *Chlamydia* species (Rockey et al., 1995); IncB and IncC were found in *C. psittaci* and *C. trachomatis*, and IncD, IncE, IncF, and IncG were found in *C. trachomatis* (Rockey et al., 2002; Kostrjukova et al., 2006). With the development of *in silico* analyses, more than 50 C*. trachomatis* and 100 C*. pneumoniae* Inc proteins have been predicted, and some of them have been verified by experiment *in vitro* (Cortes et al., 2007; Flores et al., 2007; Sixt et al., 2017; Nguyen et al., 2018).

Inc proteins have distinct coiled-coil domains that can insert into the IM and interact with host cell proteins to facilitate the exchange of materials, energy, and signals (Ronzone and Paumet, 2013). For example, IncA mediates vesicle fusion, and participates in the formation of tubule extensions *in vitro* (Weber et al., 2016). IncB interacts with host cell dynein to regulate the microtubule system (Böcker et al., 2014). CPSIT_0846 is a novel *C. psittaci* Inc protein that was identified by our team in previous work, which is the homolog of *C. psittaci* IncA (100% homology), and show 41% or 35% identity with IncA of *C. caviae* or *C. felis*. CPSIT_0846 is also a member of the transmembrane head (TMH) protein family, which shares 84% homology with the TMH family protein secreted by *C. abortus*. We previously found that CPSIT_0846 not only promoted the production of tumor necrosis factor alpha (TNF-α), interleukin (IL)-1β, and IL-6 in human monocytic THP-1 cells *in vitro*, but also induced a strong humoral and cellular immune response in mice (Wu et al., 2016; Ran et al., 2017). Therefore, we speculate that this protein may be closely related to the pathogenic mechanism of *C. psittaci*.

Apoptosis is a critical defence mechanism that helps to maintain balance in the immune system and protect hosts against pathogen infection (Naoi et al., 2019). *Chlamydia* can inhibit apoptosis in host cells during the early stages of infection to ensure their proliferation (Wen et al., 2019). *C. trachomatis* can activate PDPK1-MYC, enhance the binding of hexase II (HKII) to mitochondria, and thereby resist apoptosis (Al-Zeer et al., 2017). *C. psittaci* can inhibit HeLa cell apoptosis through the mitochondria-mediated apoptosis pathway, and this process is regulated by ERK1/2 and Bcl-2 family proteins (Li et al., 2018). Chlamydial Inc proteins also participate in the regulation of host cell apoptosis. Moore and his colleagues found Chlamydial IncA interacted with the G3BP1 partner, and affected the expression of c-myc mRNA, thereby inhibiting host cell apoptosis (Moore and Ouellette, 2014). CPSIT_0846, the homolog of *C. psittaci* IncA, can be detected at 2 h after host cell infection, and throughout the whole development cycle (Wu et al., 2016). So, CPSIT_0846 may also participates in the pathogenesis of *C. psittaci* by regulating apoptosis in host cells.

In this study, we explored the role of CPSIT_0846 in regulating host cell apoptosis, with a focus on MAPK signaling pathways. Our results clearly showed that CPSIT_0846 inhibited mitochondrial-mediated apoptosis *via* the ERK1/2 and SAPK/JNK signaling pathways in HeLa cells, which may be related to the development cycle and pathogenic mechanism of *C. psittaci*.

## Materials and Methods

### Preparation of CPSIT_0846 Recombinant Protein

Recombinant CPSIT_0846 protein was prepared as formerly described (Ran et al., 2017). Fusion proteins were purified using Ni-nitrilotriacetic acid (NTA) beads (QIAGEN Inc., Germany), and concentrated with a BCA Protein Assay Kit (Dojindo, Tokyo, Japan). Endotoxins were eliminated with a polymyxin B cartridge (Sigma, St. Louis, MO, USA) and detected using inactivated cell lysate extracted limulus blood (Chinese Horseshoe Crab Reagent Manufactory, Ltd., Xiamen, China).

### Cell Treatment

Human cervical cancer epithelial (HeLa229) cells obtained from the American Type Culture Collection were grown in 6-well, 24-well, or 96-well flat-bottom plates (Corning incorporated, Corning, NY, USA) to a suitable density, and maintained in Dulbecco’s modified minimal essential medium (DMEM; Hyclone, USA) supplemented with 10% fetal bovine serum (Gibico, NY, MA). After culturing overnight at 37°C in an incubator supplied with 5% CO_2_, the cell culture medium was replaced with serum-free DMEM. For individual experiments with or without 50 μM ERK1/2 inhibitor U0126 (Cell Signaling Technology, Beverly, MA, USA) or 30 μM SAPK/JNK inhibitor SP600125 (Sigma-Aldrich, Munich, Germany), recombinant CPSIT_0846 was added to the culture medium at different concentrations for different times. For induction of apoptosis, after a 20 h treatment with CPSIT_0846, 1 μM staurosporine (STS; Sigma-Aldrich) was added over a period of 4 h, or 10 μM carbonyl cyanide 3-chlorophenylhydrazone (CCCP; Sigma) was added for 30 min.

### CCK-8 Cell Viability Analysis

A cell counting kit-8 (CCK-8, Dojindo) was used to assay the effect of CPSIT_0846 on the viability of Hela cells. Cells were placed in 96-well plates at suitable densities and cultured overnight. After rinsing with sterile phosphate-buffered saline (PBS; Hyclone, Logan, USA), HeLa229 cells were treated with recombinant CPSIT_0846 for 24 h, and PBS alone or heated CPSIT_0846 served as controls. CCK-8 was diluted to 5 mg/ml with DMEM containing 10% fetal bovine serum, and 100 μl CCK-8 solution was added to each well. After culturing for 2 h, the optical density of each sample was measured with a microplate reader at 490 nm and cell viability (%) was calculated.

### Hoechst 33258 Staining

Sterile round glass slides were placed into a plate in advance, and HeLa cells at an appropriate density were seeded onto the glass slides and cultured overnight. After the cells were sitmulated with different concentrations of CPSIT_0846 for different times, Hoechst33258 (Sigma) staining was carried out to explore nuclear morphological changes. Specifically, after treatment, cells were fixed with 4% paraformaldehyde buffer for 30 min, then permeabilised with 0.3% (v/v) Triton X-100 for 10 min. After discarding the liquid in each well, Hoechst 33258 fluorescent staining solution was added and incubated at 37°C in the dark for 30 min. The morphology of cells in each group was observed and photographed using a TS 2R fluorescence microscope (Nikon, Tokyo, Japan). The percentage of apoptotic cells was calculated by counting cells in five random fields under the microscope.

### Flow Cytometry Assay

After treatment, cells were stained with combined annexin V-FITC/PI (KeyGEN, Nanjing, China) according to the manufacturer’s instructions. Briefly, resuspend HeLa cells were mixed with 500 μl binding buffer, followed by addition of 5 μl annexin V-FITC and 5 μl propidium iodide, and then incubated for 10 min, and the apoptotic cell population was determined by flow cytometry (Becton Dickinson, Franklin, New Jersey, USA).

### JC-1 Staining

JC-1 staining was used to assess mitochondrial membrane potential (MMP). After HeLa cells were stimulated with CPSIT_0846, 10 μM CCCP was added to each well and incubated for 30 min. MMP was measured using a JC-1 Mitochondrial Membrane Potential Detection Kit (Abcam, Cambridge, MA, USA) according to the manufacturer’s instructions. Briefly, 500 μl working solution was added to each well and incubated for 20 min at 37°C with 5% CO_2_, then washed with buffer solution. After that, cells were observed and photographed using a TS 2R fluorescence microscope (Nikon). Cells with a stable MMP displayed red fluorescence (J-aggregates), while displayed green fluorescence (monomer) with a lower MMP.

### Indirect Immunofluorescence Assay

Indirect immunofluorescence was used to analyze the release of cytochrome c (Cyt.c) into the cytoplasm. After HeLa cells were treated with CPSIT_0846, 10 μM CCCP was added to each well and incubated for 30 min. Thereafter, 4% paraformaldehyde buffer was used to fix cells for 30 min, and cells were permeabilised with 0.3% Triton X-100 for 10 min. After washing and blocking, cells were immunostained with rabbit anti-cytochrome c antibody (1: 200; Abcam) overnight at 4°C, then incubated with Cy2-conjugated goat anti-rabbit IgG (1:200; Green, Jackson ImmunoResearch Laboratories, USA) for 1 h at room temperature. DNA was stained blue with Hoechst 33258 (1:400; Sigma). Fluorescence images were visualized with a TS 2R fluorescence microscope (Nikon).

### Western Blotting Assay

Cells were collected and lysed with lysis buffer (containing protease and phosphatase inhibitors) on ice for 15 min. Lysates were collected and boiled at 100°C for 10 min. An equal amount of protein was loaded onto each lane of a 10% or 12% sodium dodecyl sulfate polyacrylamide gel electrophoresis (SDS-PAGE gel), separated by electrophoresis, and transferred to a polyvinylidene fluoride (PVDF) membrane (Millipore, Billerica, USA). Afterwards, the PVDF membrane was blocked with blocking solution (containing 5% skim milk, 0.02% Tween-20 and Tris buffer) for 2 h, and incubated overnight at 4°C with specific primary antibodies. Glyceraldehyde-3-phosphate dehydrogenase (GAPDH) was used as a control. Primary antibodies recognising GAPDH, Bax, Bcl-2, ERK1/2, p-ERK1/2, SAPK/JNK, p-SAPK/JNK, p38, p-p38, caspase-3, cleaved caspase-3, caspase-9, cleaved caspase-9, PARP, cleaved PARP rabbit mAb, and p53 mouse mAb were purchased from Cell Signaling Technology. After washing five times with PBST, the membrane was incubated with the corresponding horseradish peroxidase (HRP)-conjugated Affinipure Goat Anti-Rabbit IgG (H+L; Abcam) or HRP-conjugated Affinipure Goat Anti-Mouse IgG (H+L; Abcam) for 1 h. The ECL reaction was performed with developing solution and the reaction signal was detected by a G:BOX Chemi XXX9 system (Syngene, Cambridge, UK).

### Statistics

All experiments were performed in triplicate independently, and data are reported as mean ± standard deviation (SD). The results of western blots were scanned and analyzed by Image J (NIH Image, Bethesda, MD) and OriginPro 9.0 software. One-way analysis of variance (ANOVA) was used for comparisons between two groups. Differences were considered significant at *P* < 0.05 (*, *P* < 0.05 and **, *P* < 0.01).

## Results

### Preparation of Recombinant CPSIT_0846

**Figure 1:**
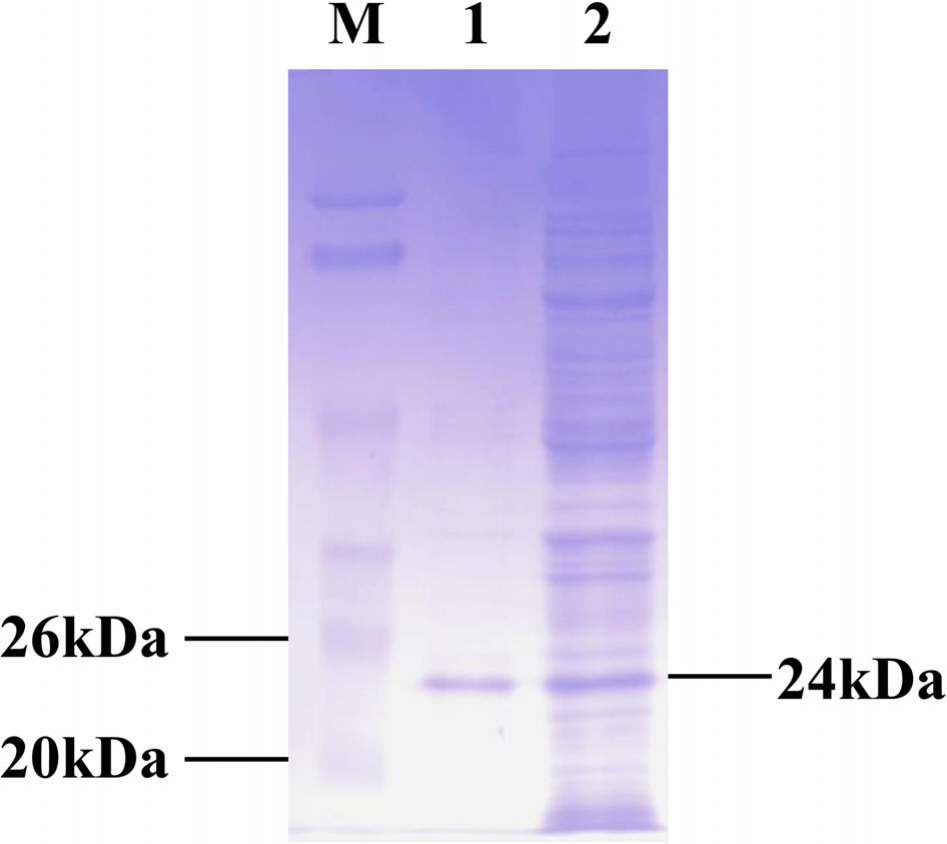
Production of recombinant CPSIT_0846. The pET-30a (+)-CPSIT_0846 plasmid was constructed and transformed into *Escherichia coli* BL21 cells, and His-tagged protein was expressed following IPTG induction. Fusion proteins were purified with Ni-nitrilotriacetic acid (NTA) beads. M, markers; 1, purified CPSIT_0846.

Recombinant CPSIT_0846 protein was expressed from the pET-30a(+)-CPSIT_0846 plasmid by isopropyl β-D-thiogalactopyranoside (IPTG) induction. Endotoxin elimination and purification resulted in recombinant CPSIT_0846 protein with a molecular weight of 24 kDa (**Figure 1**).

### CPSIT_0846 Protein Has No Significant Effect on HeLa Cell Viability

**Figure 2:**
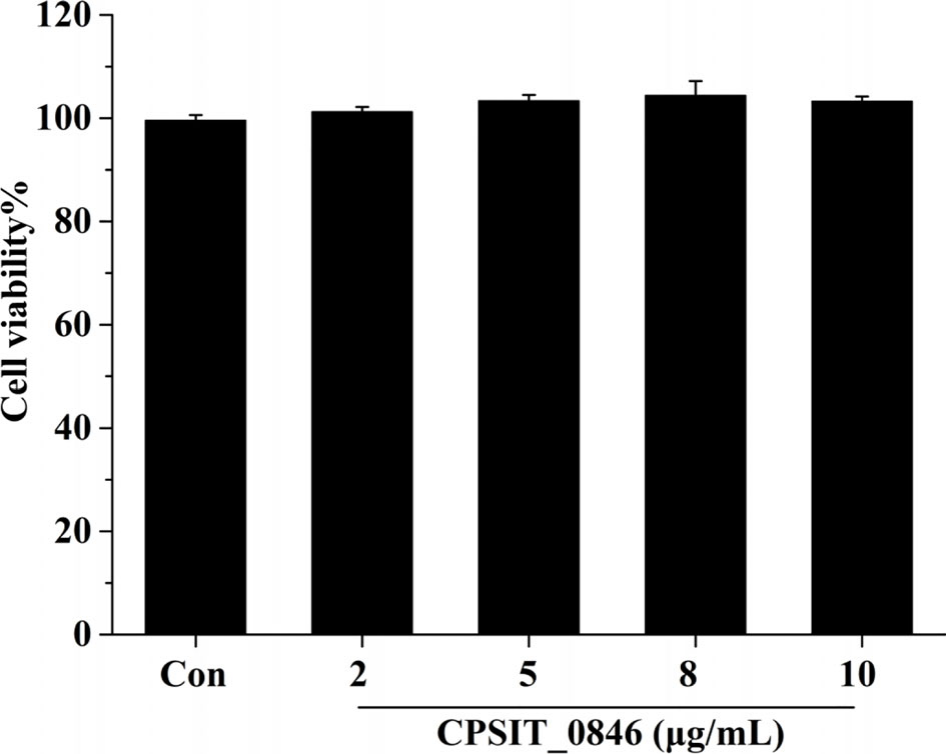
Cell viability (%) of HeLa cells after CPSIT_0846 treatment. HeLa cells were treated with recombinant CPSIT_0846 protein at concentrations of 5, 10, 15, and 20 μg/ml for 24 h, and cell viability was determined by CCK-8 assay. There were no significant differences in cell viability between CPSIT_0846 treatment and control groups (*P* > 0.05).

HeLa cells were treated with different concentrations of CPSIT_0846 protein for 24 h, and cell viability (Cell viability%) was determined using a CCK-8 cell activity detection kit. As shown in **Figure 2**, the viability of HeLa cells treated with different concentrations of CPSIT_0846 protein did not differ from that of the control group (*P* > 0.05), which indicates that recombinant CPSIT_0846 has no obvious toxic effect on HeLa cells.

### CPSIT_0846 Inhibits HeLa Cell Apoptosis

**Figure 3:**
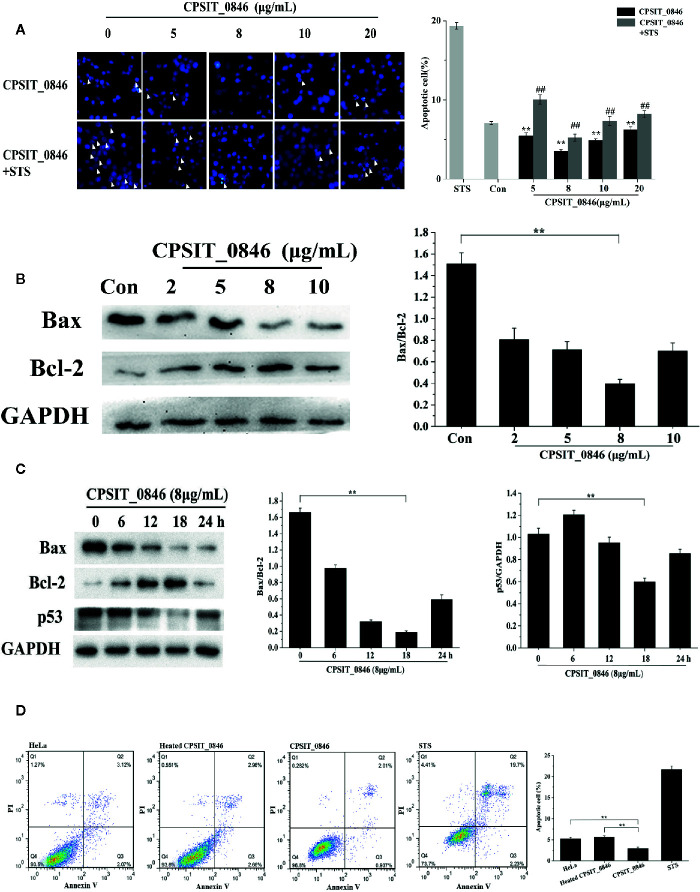
The effect of CPSIT_0846 on apoptosis. **(A)** HeLa cell apoptosis probed by Hoechst 33258 staining following treatment with different concentrations of CPSIT_0846. White arrows indicate apoptotic bodies (×400 magnification). The apoptotic rate (%) = (number of cells undergoing apoptosis in five random fields/total number of cells in five random fields) ×100%. **P < 0.01 compared with the control group; ^##^ *P* < 0.01 compared with the STS induction group. **(B)** Expression levels of Bax and Bcl-2 in HeLa cells following treatment with different concentrations of CPSIT_0846 for 24 h. **(C)** Expression levels of Bax, Bcl-2 and p53 following treatment with 8 μg/ml CPSIT_0846 for different times. GAPDH was used as a control. **(D)** HeLa cell apoptosis detected by flow cytometry. The apoptotic rate was analyzed by combined annexin V-FITC/PI staining, which showed that 8 μg/ml CPSIT_0846 protein had an obvious inhibitory effect on apoptosis following treatment for 18 h. Experiments were performed in triplicate independently (**P < 0.01).

When *C. psittaci* infects host cells, CPSIT_0846 can be detected in 2 h after *C. psittaci* infection, and throughout the subsequent infection cycle. So, CPSIT_0846 may play a role in the whole stages of infection. We first chose 24 h as the experimental time to determine the optimal concentration of CPSIT_0846. Hoechst 33258 staining showed that HeLa cells treated with CPSIT_0846 contained fewer apoptotic bodies and exhibited a lower apoptotic rate than untreated cells. After STS inducer treatment, the morphology of cells showed obvious nucleoli shrinkage or fragmentation, and numerous “bear-palm” apoptotic bodies were evident. Although apoptotic bodies were also observed in CPSIT_0846-treated cells, the morphology of most cells were normal, and the degree of apoptosis was greatly reduced (**Figure 3A**). After CPSIT_0846 treatment, the expression level of Bax was down-regulated, while Bcl-2 expression was increased, and the ratio of Bax/Bcl-2 was lower (**Figure 3B**). And the changes after 8μg/mL CPSIT_0846 treatment are the most obvious, so 8μg/mL was chosen as the optimal concentration for the next experiments.

Then, with 8 μg/ml CPSIT_0846 treatment, the Bax/Bcl-2 ratio, expression level of p53 and apoptotic rate decreased and reached the lowest values at 18 h post-stimulation. When 8 μg/ml CPSIT_0846 was applied for 18 h, the ratio of Bax/Bcl-2 and the expression level of p53 were strongly down-regulated (**Figure 3C**). Cell apoptosis was further confirmed by combined annexin V-FITC/PI staining, and the apoptotic rate of the control group was 5.19%. After STS inducer treatment, the apoptotic rate was increased significantly (21.93%), demonstrating that the STS inducer could effectively induce cell apoptosis. The apoptotic rate (2.95%) in the CPSIT_0846 treatment group was significantly lower than that in the control group, but the apoptotic rate (5.62%) of the group given heated CPSIT_0846 protein was similar to that of the control group (**Figure 3D**). These results indicate that CPSIT_0846 inhibits apoptosis in HeLa cells.

### CPSIT_0846 Facilitates Resistance to Apoptosis Involving the Mitochondrial Apoptotic Pathway

**Figure 4:**
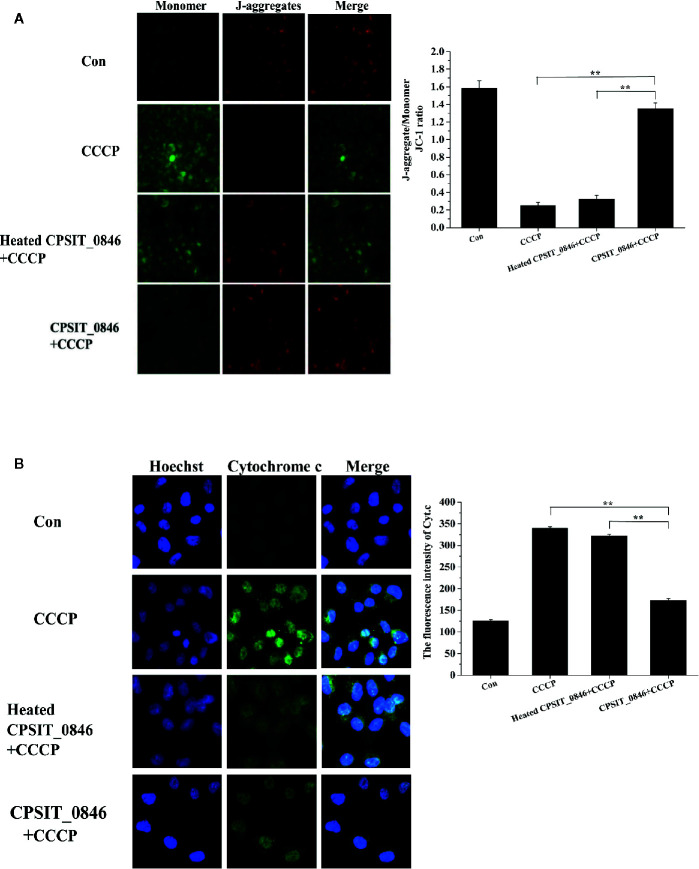
The effect of CPSIT_0846 protein on mitochondrial apoptosis. HeLa cells were treated with CCCP (10 μM) inducer after CPSIT_0846 (8 μg/ml) treatment for 18 h. **(A)** Changes in mitochondrial membrane potential in HeLa cells detected by JC-1 staining under a TS 2R fluorescence microscope (×400 magnification). There were significant differences in mitochondrial membrane potential following CCCP induction among CPSIT_0846-treated, CCCP-treated, and heat-inactivated CPSIT_0846 groups. **(B)** Changes in Cyt.c in HeLa cells detected by indirect immunofluorescence (×400 magnification). Cyt.c was visualized by Cy2 (green) and DNA was stained by Hoechst (blue). Experiments were performed in triplicate independently, and data were represented as means ± SD (**P < 0.01).

The effects of CPSIT_0846 on mitochondrial membrane potential (MMP) and cytochrome c (Cyt.c) release from HeLa cells were evaluated by JC-1 fluorescence staining and indirect immunofluorescence assay. In the control group, the fluorescence signal was mainly red (J-aggregates), indicating the cells could maintain a stable MMP. However, in the CCCP treatment group the fluorescence signal was green (monomer), indicating a reduction in MMP. After CCCP treatment, cells in the CPSIT_0846 treated group displayed a stronger red fluorescence signal (J-aggregates), and the ratio of red-green fluorescence signal in the JC-1 assay was significantly increased, which indicates that the MMP was stable. However, the MMP of cells treated with CCCP alone or heated CPSIT_0846 protein was significantly decreased (**Figure 4A**). In addition, we also found that after treated with CCCP, mitochondria-derived Cyt.c was detected in the cytoplasm in cells exposed to heated CPSIT_0846, but there was no significant change in the CPSIT_0846 treated group (**Figure 4B**). The above results indicate that CPSIT_0846 inhibits apoptosis *via* the mitochondrial pathway.

### CPSIT_0846 Inhibits Apoptosis *via* the ERK1/2 and SAPK/JNK Signaling Pathways

**Figure 5:**
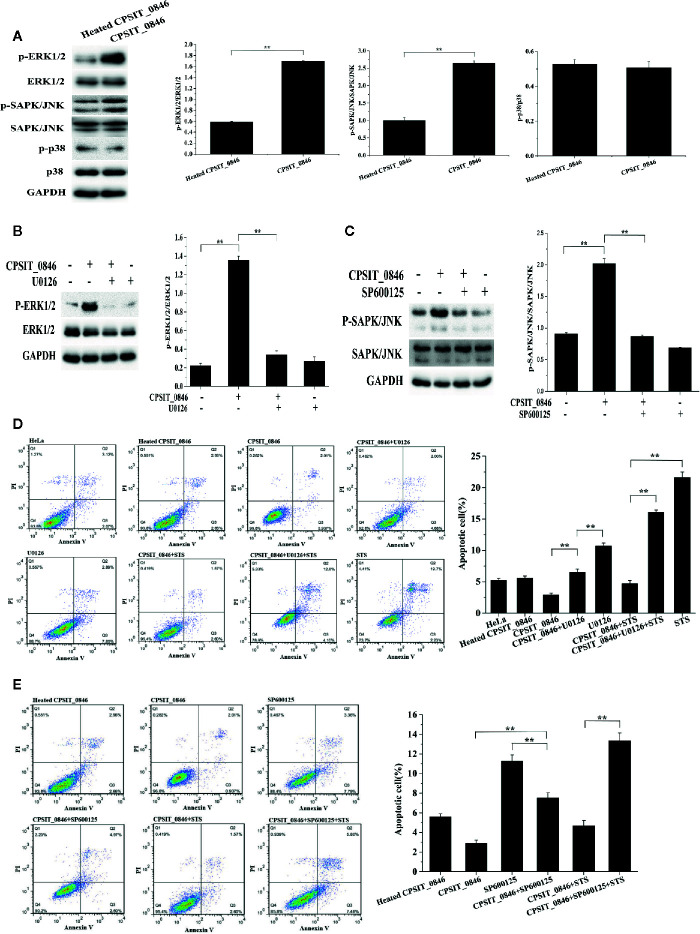
ERK1/2 and SAPK/JNK signaling pathways regulate CPSIT_0846-mediated resistance to host cell apoptosis. **(A)** CPSIT_0846 activated ERK1/2 and SAPK/JNK, but not p38. **(B, C)** Phosphorylation of ERK1/2 or SAPK/JNK was inhibited by 30 μM U0126 or SP600125. GAPDH was used as a control. **(D, E)** Inhibition of apoptosis by CPSIT_0846 protein is weakened following treatment with U0126 or SP600125. Experiments were performed in triplicate independently (**P <0.01).

After treating with CPSIT_0846 for 18 h, the levels of phosphorylated ERK1/2 and SAPK/JNK, but not p38, were significantly increased and could be significantly inhibited with 30 μM U0126 (ERK1/2 inhibitor) or SP600125 (SAPK/JNK inhibitor) (**Figures 5A–C**). Simultaneously, annexin V-FITC/PI staining results showed that after ERK1/2 or SAPK/JNK was inhibited, the apoptosis rates of CPSIT_0846-treated cells were significantly increased with or without STS treatment (**Figures 5D, E**). These results indicate that ERK1/2 and SAPK/JNK signaling pathways are involved in regulation of host cell apoptosis by CPSIT_0846.

**Figure 6:**
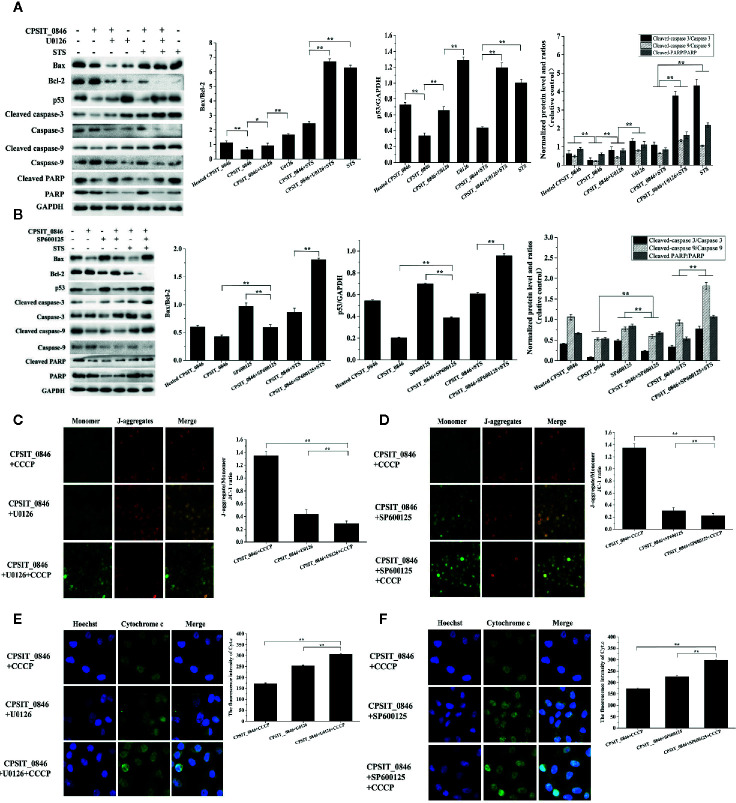
Effect of CPSIT_0846 on host cell mitochondrial apoptosis following inhibition of ERK or SAPK/JNK signalling pathways. **(A, B)** The expression levels of Bax, Bcl-2, and p53, cleaved caspase-3, cleaved caspase-9, and cleaved PARP by western blotting following treatment with U0126 or SP600125. GAPDH was used as a control. **(C, D)** Changes in MMP detected by JC-1 staining (×200 magnification). **(E, F)** Cyt.c detection by indirect immunofluorescence. Experiments were performed in triplicate independently. Data were represented as means ± SD (*P < 0.05; **P < 0.01).

Next, the expression levels of Bax, Bcl-2, p53, caspase-3, caspase-9, and PARP were analyzed by western blotting. Compared with cells exposed to heated CPSIT_0846, cells treated with intact CPSIT_0846 displayed a lower Bax/Bcl-2 ratio, and the expression levels of p53, cleaved caspase-3, cleaved caspase-9 and cleaved PARP were lower. However, after the ERK1/2 or SAPK/JNK signaling pathways were inhibited in CPSIT_0846-treated HeLa cells, both with and without STS induction, the ratio of Bax/Bcl-2 and expression levels of p53, cleaved caspase-3, cleaved caspase-9 and cleaved PARP were significantly up-regulated (**Figures 6A, B**), while MMP was decreased (**Figures 6C, D**), and Cyt.c was discharged into the cytoplasm (**Figures 6E, F**). These results indicate that the CPSIT_0846 protein may regulate host cell apoptosis through the mitochondrial pathway, and this process is related to ERK1/2 and SAPK/JNK signaling pathways.

## Discussion

*Chlamydia* are obligate intracellular parasitic microorganisms with an unique development cycle. *C. psittaci* is capable of cross-host transmission between a range of mammalian hosts including humans (Branley et al., 2016). *Chlamydia* can secrete effector proteins *via* the type 3 secretion system (T3SS) and inject them into host cells to assist survival (Parrett et al., 2016; Jelocnik et al., 2017). However, the mechanism used by *C. psittaci* to evade the host immune system and the mechanism of pathogenicity remains unclear.

A complete chlamydial development cycle is usually 36 to 72 h (Harkinezhad et al., 2009). When *Chlamydia* invades the host cells, it replicates within the inclusion, a membrane-bound vacuole, and alternates between elementary bodies (EBs) and reticulate bodies (RBs). RBs re-differentiate into offspring EBs. Newly formed EBs are released by host cell lysis, and then infect neighboring cells, initiating a new replication cycle (Dautry-Varsat et al., 2005). In the development cycle, *Chlamydia* produces a large number of effector proteins, some of which can inhibit host cell apoptosis to maintain a suitable living environment and help them obtain nutrients. When the new generation of EBs is formed, some Chlamydial effector proteins can promote host cell apoptosis, such as CPSIT_0959 in *C. psittaci*, which helps the release of offspring EBs, and ultimately promotes EBs to infect neighboring host cells (Sixt and Kroemer, 2017; Sun et al., 2017; Weber et al., 2017; Wen et al., 2019; Marschall et al., 2020). Whether they are to promote or inhibit host cell apoptosis, all of these are the way that *Chlamydia* has evolved in order to survive in the host cell.

Inc proteins are located in the *Chlamydia* inclusion membrane (IM), where they promote its fusion and assist the absorption of nutrients, help resist host cell autophagy, and interfere with intracellular signaling (Mital et al., 2015; Weber et al., 2017; Almeida et al., 2018). *Chlamydia* can encode different Inc proteins during different phases of the infection cycle, which help *Chlamydia* to escape host immune system (Kostriukova et al., 2008). For example, the major Inc proteins encoding genes of *C. trachomatis* are early genes, and IncD, IncE, IncF, IncG, CT228, and CT229 were expressed in the first few hours after chlamydial infection (Shaw et al., 2000; Scidmore and Hackstadt, 2001); Cpn0712 and Cpn0703 were secreted into the inclusion membrane at 20 h after *C. pneumoniae* infected host cells for 20 h (Vandahl et al., 2002).

Previous studies have shown that some Inc proteins can inhibit host cell apoptosis, which is essential for *Chlamydia* infection (Li et al., 2018). *C. trachomatis* IncG interacts with the mammalian 14-3-3β protein in HeLa cells, and 14-3-3β in turn recruits Bad (a pro-apoptotic protein) to the chlamydial inclusion complex, resulting in inhibition of host cell apoptosis (Scidmore and Hackstadt, 2001). In our study, combined Hoechst 33258 and annexin V-FITC/PI staining analysis showed that the CPSIT_0846 protein could significantly reduce the apoptotic rate in HeLa cells, and decrease the Bax/Bcl-2 ratio, which indicates that it inhibits host cell apoptosis.

The tumor suppressor p53 is an important transcription factor with multiple regulatory functions, including regulating DNA repair, controlling cell metabolism and cell cycle arrest, and governing cell apoptosis (Sun et al., 2018; Li et al., 2019). Expression level of p53 is regulated by Bcl-2 family genes. For example, up-regulation of Bax and other apoptotic proteins can stimulate the expression of p53, triggering the collapse of MMP, which causes the release of Cyt.c and other mitochondrial components into the cytoplasm, followed by caspase-9 and caspase-3 activation, eventually leading to cell apoptosis (Lebedeva et al., 2003; Yeh et al., 2009). Our result showed that p53 expression was significantly down-regulated in HeLa cells treated with CPSIT_0846, consistent with CPSIT_0846 inhibiting host cells apoptosis.

A decrease in MMP is a characteristic of the early stages of mitochondrial- dependent apoptosis, in which the up-regulation of the Bax/Bcl-2 ratio causes Cyt.c release into the cytoplasm, and then Caspase-9 and Caspase-3 are activated, accompanied by the lysis of PARP, which eventually leads to cell apoptosis (Bi et al., 2018; Ye et al., 2019). *Chlamydia* can maintain MMP stability, which might be inseparable from the mechanisms of persistent infection and resistance to host cell apoptosis (Li et al., 2018). CPSIT_0846 can prevent CCCP-induced mitochondrial membrane potential reduction and inhibit the release of Cyt.c into the cytoplasm, which indicates that CPSIT_0846 may inhibit host cell apoptosis by acting on mitochondrial-mediated signaling pathways.

Mitogen-activated protein kinases (MAPKs) form the upstream signaling pathway of mitochondria, including three important subfamilies (ERK1/2, SAPK/JNK, and p38), which can be activated in response to various extracellular stimuli (Krüll et al., 2004; Papa et al., 2019). ERK1/2 are activated by MAP/ERK1/2 kinases 1 and 2 (MEK1/2) by dual phosphorylation at their threonine 202 and tyrosine 204 residues (Cagnol and Chambard, 2010). Activated ERK1/2 and SAPK/JNK can directly phosphorylate a variety of cytoplasmic and nuclear substrates involved in various cellular processes such as proliferation, differentiation, apoptosis and survival (He et al., 2015; Meng et al., 2019; Sharma et al., 2019). p38 protein kinase is a tyrosine phosphoprotein kinase associated with inflammation and growth factor responses (Mo et al., 2020). When exploring the role of MAPK signaling pathways in CPSIT_0846-mediated regulation of host cell apoptosis, we found that CPSIT_0846 activated ERK1/2 and SAPK/JNK but not p38 in HeLa cells. After inhibiting ERK1/2 or SAPK/JNK with U0126 or SP600125, the ability of CPSIT_0846 to inhibit apoptosis in HeLa cells was limited, and the Bax/Bcl-2 ratio and expression level of p53 were significantly elevated. BCL-2 family members maintain mitochondrial integrity, and are the main regulators for activation ofcaspase proteins. When mitochondria receive multiple apoptotic signals, the MMP drops and Cyt.c is released from the intermembrane space, causing a series of reactions that eventually lead to caspase activation (Cheng et al., 2020). After the ERK1/2 or SAPK/JNK signaling pathway was inhibited, CPSIT_0846 decreased the MMP, but increased Cyt.c release, and the abundance of cleaved caspase-3, cleaved caspase-9, and cleaved PARP was significantly increaseed regardless of STS induction. Therefore, the inhibitory effect of CPSIT_0846 on apoptosis may be partly due to its ability to activate ERK1/2- and SAPK/JNK-mediated mitochondrial pathways.

*Chlamydia* can deliver inclusion membrane proteins from their intracellular niche to the host cytoplasm mainly through type 3 secretion system. Some domains of these secreted inclusion membrane proteins may expose into the cytoplasm and combine with molecular chaperone proteins to promote folding or delivery, ultimately achieve the purpose of manipulating host cells (Weber and Faris, 2018). The hypothetical inc protein CPSIT_0846 used in this study is a supernatant protein expressed by a prokaryotic expression system, which usually does not interfere with the native conformation of the protein (Kesidis et al., 2020). But the mechanism of how CPSIT_0846 enter host cells and which domains are exposed into the cytoplasm and which molecular chaperones are bound with them are still unclear. And these complicated mechanisms are exactly what we prepare to study further.

## Data Availability Statement

The original contributions presented in the study are included in the article/supplementary material. Further inquiries can be directed to the corresponding authors.

## Author Contributions

LilC, HW, and TT conceptualized the experiments and methods. XC and LiC contributed to cell experiments. TT and LL carried out the Western blotting and fluorescent staining experimentations. LilC, QB, and YC contracted the data collation and statistical analysis. TT and HW wrote the manuscript. LilC, ZL, and YC joined for language editing and reviewing of the manuscript. All authors contributed to the article and approved the submitted version.

## Funding

This work was supported by the National Natural Science Foundation of China (grant no. 31600150, 81572011), the Natural Science Foundation of Hunan Province (2020JJ4527, 2019JJ50494), the Scientific Research Fund of Hunan Provincial Education Department (20A438), and the Fund of Hunan Key Laboratory (2019TP1027).

## Conflict of Interest

The authors declare that the research was conducted in the absence of any commercial or financial relationships that could be construed as a potential conflict of interest.
